# Bibliometric insights into breast cancer organoid chips: trends and emerging areas – a commentary

**DOI:** 10.1097/JS9.0000000000003512

**Published:** 2025-09-17

**Authors:** Zengwei Kou

**Affiliations:** Department of Laboratory Medicine and Pathobiology, Temerty Faculty of Medicine, University of Toronto, Toronto, ON, Canada

*Dear Editor,*

I read with great interest the article entitled “Bibliometric insights into breast cancer organoid chips: trends and emerging areas”[1] recently published in International Journal of Surgery, as well as the accompanying commentary[2]. Together, these two papers provide valuable bibliometric perspectives for understanding the applications and emerging frontiers of organoids in breast cancer research. I especially appreciate the original article’s comprehensive summary of the history, development, and frontiers of organoid technology. At the same time, I partially agree with the commentary, which highlighted potential issues in the original retrieval strategy.

Specifically, the original Boolean search appeared overly complicated and retrieved a considerable number of irrelevant publications, likely due to the inclusion of Web of Science Keyword Plus terms – terms automatically generated from cited references but not necessarily present in the article itself. Based on both the authors’ methodology and our understanding, I adopted an improved search strategy and performed cross-database retrieval across PubMed, Scopus, and Web of Science^[3,4]^. In contrast to the commentary, I did not include CNKI databases, as in practice Chinese researchers tend to publish high-quality biomedical research in English journals indexed by the three aforementioned databases.

At the time of my search, after de-duplication and manual validation, I identified 199 publications, exceeding the 161 articles reported in the original study (Fig. 1A). My bibliometric analysis, conducted in strict accordance with the TITAN guidelines[5], yielded results broadly consistent with the original report but provided more nuanced insights. I found that the cumulative number of publications followed an exponential growth model (*R*^2^ = 0.975) (Fig. 1B), and that the United States, rather than China, was the leading country in publication output (Fig. 1C). Moreover, the most productive institution was PCSHE, not Fudan University (Fig. 1D). As in the original article, *Lab on a Chip* and *Cancer Research* remained central in the journal co-citation network (Fig. 1E). A three-field plot further revealed that influential authors predominantly focused on “microphysiological systems” and the “tumor microenvironment” (Fig. 1F), and keyword co-occurrence analysis confirmed these findings, showing that “tumor microenvironment,” “microphysiological system,” and “breast cancer” occupied central positions (Fig. 1G) – contrasting with the emphasis on “expression” and “metastasis” in the original article. Finally, keyword trend analysis demonstrated rapid growth in terms such as “tumor microenvironment,” “breast cancer,” and “metabolism,” underscoring these as key emerging frontiers (Fig. 1H).

**Figure 1.**
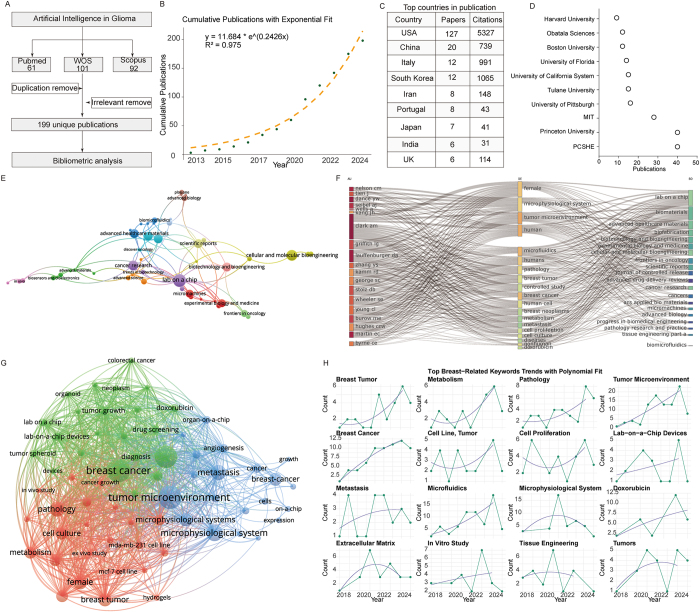
Bibliometric landscape of research on breast cancer organoid chips. (A) Search strategy employed for literature retrieval. (B) Cumulative publication output and itsfitting trend. (C) Leading countries ranked by publication volume. (D) Top contributing institutions. (E) Co-citation network of core journals. (F) Three-field plot linking key authors, keywords, and key journals. (G) Keyword co-occurrence network highlighting major research clusters. (H) Temporal trends of top breast-related keywords with polynomial fitting, illustrating emerging research directions.

I also wish to correct the interpretation of Figure 6 in the original article: the figure does not represent a “citation relationship,” but rather reflects logical grouping and topic evolution.

In summary, by employing an optimized cross-database retrieval strategy, I refined and extended the bibliometric insights of the original article. I believe this approach better captures the evolving research landscape of organoid technology in breast cancer studies.

## Data Availability

No dataset was generated in this study.

## Declarations

No AI was used in this paper.
